# Assisting Home-Based Resistance Training for Normotensive and Prehypertensive Individuals Using Ambient Lighting and Sonification Feedback: Sensor-Based System Evaluation

**DOI:** 10.2196/16354

**Published:** 2020-06-29

**Authors:** Mustafa Radha, Niels den Boer, Martijn C Willemsen, Thom Paardekooper, Wijnand A IJsselsteijn, Francesco Sartor

**Affiliations:** 1 Royal Philips Eindhoven Netherlands; 2 Eindhoven University of Technology Eindhoven Netherlands; 3 The Hague University of Applied Sciences The Hague Netherlands; 4 Bangor University Bangor United Kingdom

**Keywords:** hypertension, sonification, respiratory guidance, intrinsic motivation, physical exertion

## Abstract

**Background:**

Physical exercise is an effective lifestyle intervention to improve blood pressure. Although aerobic sports can be performed anywhere, resistance exercises are traditionally performed at the gym; extending the latter to the home setting may promote an increase in the number of practitioners.

**Objective:**

This study aims to evaluate a sensor-based system that guides resistance exercises through ambient lighting and sonification (A/S) feedback in a home setting in 34 study participants who were normotensive and prehypertensive.

**Methods:**

Participants took part in a 1.5-hour exercise session in which they experienced the A/S feedback (ie, experimental condition) as well as a control condition (ie, no feedback) and a reference condition (ie, verbal feedback through a human remote coach). The system was evaluated for improving exercise form (range of motion, timing, and breathing patterns) as well as psychophysiological experience (perceived exertion, attentional focus, competence, and motivation).

**Results:**

A/S feedback was significantly better than the control for concentric (mean 2.48, SD 0.75 seconds; *P*<.001) and eccentric (mean 2.92, SD 1.05 seconds; *P*<.001) contraction times, concentric range of motion consistency (mean 15.64, SD 8.31 cm vs mean 17.94, SD 9.75 cm; *P*<.001), and perceived exertion (mean 3.37, SD 0.78 vs mean 3.64, SD 0.76; *P*<.001). However, A/S feedback did not outperform verbal feedback on any of these measures. The breathing technique was best in the control condition (ie, without any feedback). Participants did not show more positive changes in perceived competence with A/S feedback or verbal feedback.

**Conclusions:**

The system seemed to improve resistance exercise execution and perception in comparison with the control, but did not outperform a human tele-coach. Further research is warranted to improve the breathing technique.

## Introduction

### Background

Hypertension, or high blood pressure (BP), is a key risk factor for cardiovascular diseases [1]. Effective hypertension management is therefore a major theme in public health. Besides BP medication, nonpharmacological lifestyle interventions have been proven to be successful in the management of hypertension [2]. Appropriate lifestyle modifications may not only lower or control BP in patients with hypertension but also effectively delay or prevent hypertension in nonhypertensives [3]. The European Societies of Hypertension and Cardiology endorse a wide variety of lifestyle interventions for the reduction of BP: salt restriction; moderation of alcohol consumption; a diet rich in vegetables, fruits, and low-fat dairy products; weight reduction; regular exercise; and smoking cessation [3,4].

Physical exercise is a particularly effective intervention to combat hypertension [2]. Dynamic resistance training is often recommended as a supplement to aerobic exercise as several meta-analyses have concluded that it reduces BP by 2 to 3 mm Hg among people with hypertension [5]. Although such an exercise prescription can result in a positive effect on BP postexercise, during the activity itself, there is an acute heightened BP response. Sorace et al [6] identified several factors that influence the acute BP response during resistance training. It was found that the amount of cardiovascular stress is a function of load, number of sets and repetitions, contraction time, rest periods, and whether one performs the Valsalva maneuver (ie, attempt to exhale while the airway is blocked). In accordance with these findings, the American College of Sports Medicine (ACSM) guidelines on resistance exercise states that such exercises should be executed with proper form and technique to ensure optimal health benefits and reduce the risk of injuries. Movements should be rhythmic, performed at a moderate repetition duration (3 seconds concentric and 3 seconds eccentric), with a full range of motion and a normal breathing pattern without breath-holding [7]. These recommendations may be difficult to follow for inexperienced exercisers.

In addition, people who have hypertension find it hard to adhere to exercise recommendations in general [8]. Adopting a more active lifestyle often requires a difficult behavior change. Numerous barriers exist that may influence participation in physical activity [9]. Whereas aerobic activities can be performed anywhere, resistance training is traditionally performed at the gym, which a considerable number of patients with hypertension are known not to attend [10]. Encouraging people to perform resistance training at home could eliminate this barrier. Thus, tools to assist in home-based exercise could also reduce the barriers that patients with hypertension face when changing their behavior.

Research in the field of sonification has shown that movements in physical activity can be improved by providing auditory nonspeech feedback based on sensor data. Sonification, a subtype of auditory displays, covers the technique of rendering sound in response to data and interactions [11]. Kramer et al [12] defined sonification as the transformation of data relations into perceived relations in an acoustic signal for the purpose of facilitating communication or interpretation. Applications of sonification in physical activity often used the parameter mapping approach, where movement kinematics are mapped to sound parameters to inform people about their performance. Real-time sonic feedback then aims to correct and optimize one’s technique during a specific exercise. For example, Schaffert and Effenberg [13] created a sonification feedback system called Sofirow for elite rowers to enhance their perception of movement execution. Furthermore, Smith and Claveau [14] investigated how sonification can support a student in imitating the complex motion of an instructor, increasing both spatial and temporal accuracy. Yang and Hunt [15] developed a real-time sonification system to support people performing a bicep curl. They used Microsoft Kinect to track the vertical position of the hand.

Furthermore, literature in the domain of paced breathing suggests that visual stimuli can be used to guide people’s respiration [16,17], which could potentially be used to obtain a proper breathing technique during exercise. This could potentially prevent the BP–elevating Valsalva maneuver [18], which for the most part consists of holding one’s breath. Correct breathing during resistance exercise is not always intuitive to people, as some are inclined to hold their breath when lifting weight. Therefore, it is important to search for a way in which people can be supported with a proper breathing technique without breath-holding during home-based resistance training.

### Objectives

In this study, we aimed to understand whether a combination of ambient lighting and sonification (A/S) feedback could help people in need of resistance exercise in performing such exercises in a safe and effective manner. An A/S feedback system was developed for this purpose and was tested by a group of volunteers who were prehypertensive and normotensive. The effects of the system were measured using metrics of proper exercise performance as well as self-reported psychophysiological measures. These end points were compared with a control condition in which the system was not used as well as to a reference condition where a human provided tele-coaching, representing an upper bound of exercise guidance.

## Methods

### Conditions

#### Ambient Lighting and Sonification Feedback Condition

The A/S feedback system was developed such that the ambient light and sonification feedback could be delivered automatically based on recorded movement features (described in the *Movement Features* section). At the start of this condition, participants were given a short trial where they could experience how the sound changed based on their movement. Both verbal instructions and sounds of the ambient light sonification feedback were delivered through headphones.

#### Sonification

The sonification system used a change in the sound pitch to convey information about whether the user’s pace was correct, too fast, or too slow. This perceptual dimension of sound corresponds to the physical dimension known as frequency. The repetitive movement of resistance training closely resembles a sine wave. The optimal reference sine wave was calculated based on ideal contraction time lengths (3 seconds concentric and 3 seconds eccentric), and its phase was aligned with the measured movements of the participants. In this way, when a user makes a correction in his or her pace, this was almost immediately reflected in the sound pitch. The difference in movement velocity between the sinusoidal model and the measured motion was mapped to perceivable sonic frequencies. A piano sample was looped that comprised a melody in the frequency range of 185 to 247 Hz. If the obtained difference exceeded the predefined bounds, it was scaled linearly to control the transpose dial to go up or down one octave at most. This corresponds to a range with a minimum frequency of 92 Hz and a maximum frequency of 494 Hz. On reaching the concentric or eccentric end points, the time difference between the ideal end point time and actual end point time was calculated. Earcons were used to provide feedback, which are brief sounds that represent specific events or convey specific information (ie, the auditory counterpart of an icon). A *success* earcon was triggered in the case of a correct movement, and a *corrective* earcon was played to inform the user they went too far. If the participant does not reach the ideal end point, no sound is played. The earcons had a higher pitch for concentric end points compared with the eccentric earcons. In addition to assisting with a proper range of motion, an additional earcon was used that signaled the 10 repetitions mark. Before the beginning of the study, participants were familiarized with the auditory cues to ensure that the equipment was functioning properly and that they were able to hear the auditory cues.

#### Ambient Light

**Figure 1:**
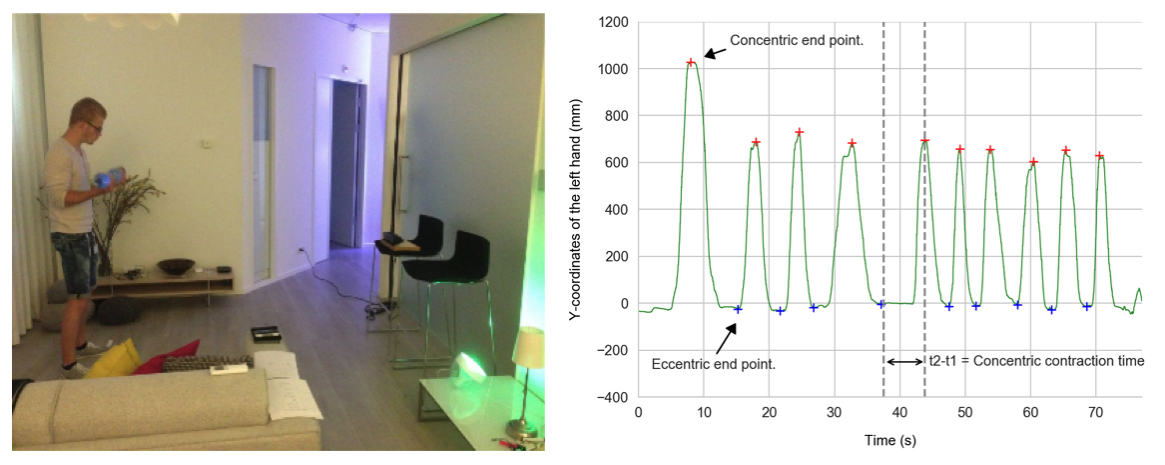
Left: example of the experimental setting while performing frontal shoulder raises showing the Kinect placement, the ambient lights (green light on the table, and a spot light on the roof, not visible), and the white panel behind which the coaching experimenter is sitting. In the verbal and A/S condition, the participant wears headphones to add the auditory feedback. Right: recording of the bicep curl with the Kinect in Max 7, showing how the concentric and eccentric contraction times and endpoints are derived. A/S: ambient light and sonification.

Three Philips hue lights were used to provide respiratory guidance (Figure 1). The lights were programmed to switch from minimal brightness to maximum brightness in 3 seconds, and vice versa. When no respiration was measured through the microphone for 6 seconds, the lights were turned off to signal the participant to resume breathing, as a means to counteract the Valsalva maneuver.

#### Control Condition: No Feedback

In the control condition, exercise performance was measured without giving feedback to participants about their performance. This meant that participants were left on their own to carry out the exercise in line with previous instructions on contraction time, range of motion, and breathing technique. In addition, they also had to count the repetitions themselves to ensure that they performed 10 repetitions as they did not receive any notification in this condition when they finished a set.

#### Reference Condition: Verbal Feedback

In the verbal condition, an experimenter was designated as the coach. The coaching experimenter first calibrated the system according to the actual range of motion of the participants. During the exercise itself, an opaque screen was placed between the coaching experimenter and the participant, simulating a tele-coach setting (Figure 1). Feedback was provided through verbal prompts, such as “Try not to hold your breath,” “Next time you may go a little higher,” “Highest point is correct,” “Try to move a bit slower,” and “The pace is good.” The experimenter made use of the metrics displayed at the maximum interface to determine what type of feedback to give.

### Study Design and Participants

#### Recruitment and Exclusion

The experiment was ethically approved by the Internal Committee for Biomedical Experiments of Philips Research in conformity with the Declaration of Helsinki. A total of 37 participants were recruited by an external recruitment agency that identified eligible volunteers based on the following inclusion criteria: (1) men and women who were normotensive, prehypertensive, or regulated stage 1 hypertensive (ie, normal BP because of medications); (2) sedentary lifestyle; (3) physically capable of exercising with the upper limbs at a moderate intensity (ie, no injuries or movement impairments); and (4) aged between 40 and 60 years and BMI<30. Furthermore, people who had chronic conditions other than regulated stage 1 hypertension, took medication other than BP–lowering medication for stage 1 hypertension, were pregnant, or had a hearing impairment were excluded. Technical difficulties on the first day of testing resulted in data loss of the first 3 participants, leaving us with data from 34 participants altogether, comprising 16 men and 18 women.

#### Study Design

A within-subjects design was used for this experiment. Participants performed 3 exercises corresponding to the 3 different conditions: control (ie, no feedback), verbal feedback, or A/S feedback. The order of feedback type, as well as the order of exercise type, were counterbalanced to cancel out fatigue, practice, and carryover effects. On the basis of the 3 conditions and 3 kinds of exercise (bicep curls, frontal shoulder raises, and inclined pectoral flies), 9 randomization blocks (3×3) were created, and the 34 participants were split into groups of 4 per block (Multimedia Appendix 1). The main dependent variables that were measured included variables related to exercise performance (ie, concentric and eccentric contraction time, concentric and eccentric end points, and respiration) and several psychological variables (ie, perceived competence, interest and enjoyment, attention, and rate of perceived exertion).

Of the 37 participants who volunteered to take place in this study, 23 participants were prehypertensive: people with baseline BP>120/80 mm Hg, of whom 17 had baseline BP>130/90 mm Hg. The rest (n=14) were normotensive (baseline BP<120/80 mm Hg). Regarding educational level, 8 participants completed secondary school, 9 secondary vocational education, 16 higher professional education, and 4 university education. Participants’ anthropometric and physiological characteristics are shown in Table 1.

**Table 1:** Characteristics of the participants after excluding 3 participants.

Characteristics	Values (N=34^a^; 16 men and 18 women), mean (SD)
Age (years)	51.11 (5.75)
Height (m)	1.75 (0.08)
Weight (kg)	76.95 (13.28)
BMI (kg/m^2^)	25.06 (3.21)
Resting systolic blood pressure (mm Hg)	129.52 (11.56)
Resting diastolic blood pressure (mm Hg)	81.85 (8.36)
Resting heart rate (bpm^b^)	70.97 (9.69)

^a^21 prehypertensive and 13 normotensive.

^b^bpm: beats per minute.

#### Power

A priori sample size calculation with the G*Power software indicated for a repeated measures design that a sample size of 36 was required to be 90% certain of detecting a medium effect size (Cohen *f*=0.25) in the concentric or eccentric contraction times (further details in the *Movement Features* section), with an alpha error of *P*<.05. Owing to double scheduling, 1 extra participant was tested. This resulted in a total sample size of 37, comprising 16 men and 21 women, with an average age of 50.97 (SD 5.77) years. However, after excluding the first 3 participants because of technical difficulties with the setup, only 34 remained.

### Measures

#### Movement Features

**Figure 2:**
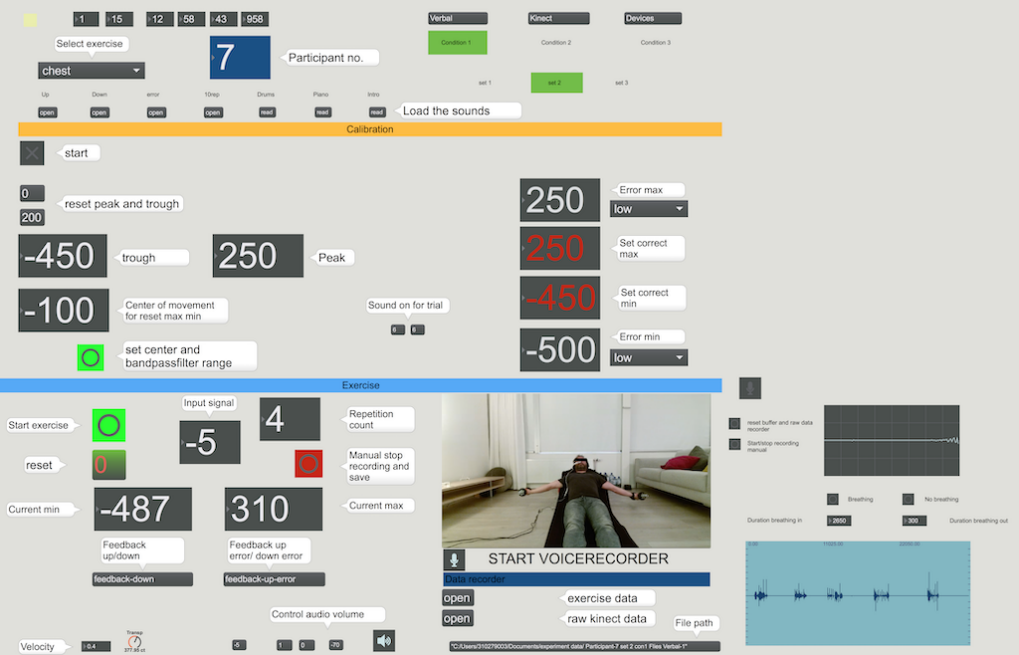
Max 7 dashboard to control experimental conditions, calibrate performance measures, and monitor exercise performance.

Spatial and temporal kinematic exercise information was captured with a depth camera, Microsoft Kinect version 2.0 (Microsoft Corporation). The camera stream was captured in Max 7 software (Cycling ’74), a visual programming language for prototyping interactive multimedia applications. The Max plug-in dp.kinect2 was used [19], with which the 3D coordinates of the limb’s joints can be extracted. As the resistance training exercises in this study all involved congruent arm movements, only the y-coordinates of the left hand were used. These coordinates were recorded at 30 frames per second. To deal with measurement errors and noise in the signal, the coordinate signal was smoothed with the dp.kinect smoothing filter (dp.kinect2 @smoothing 0.5 0.8 0.3 0.01 0.01). The main interface was created to facilitate nonautomated interaction between the experimenter and the system, such as setting the condition and a set number for an exercise session. Finally, exercise quality–related metrics were visualized to help the researcher (who was acting as a tele-coach) to assess participants’ performance during the verbal feedback condition (Figure 2).

The turning points from the lifting phase to the lowering phase, and vice versa, represented the concentric and eccentric end points, which were used to assess whether participants were able to exercise with a range of motion that corresponded to what was instructed (Figure 1). In the case of bicep curls and frontal shoulder raises, the distance of the hand, measured in millimeters, was taken relative to the person’s center of mass. For the pectoral flies, the person’s head was taken as the origin, as this was the only stable reference point Kinect could detect while laying down in an inclined position. Each repetition was divided into a lifting phase (ie, concentric contraction time) and a lowering phase (ie, eccentric contraction time) to determine if participants were able to maintain the instructed exercise pace, either with or against gravity (Figure 1). Concentric and eccentric contraction times were defined as the time change (delta) between a concentric and an eccentric end point. In line with the ACSM guidelines for resistance training and in consultation with a fitness coach, participants were instructed to exercise at a pace of 3 seconds up and 3 seconds down. The participants were instructed to perform exactly 10 repetitions. In both the verbal and A/S conditions, people were informed when they reached that number, but in the control condition, participants had to count by themselves.

#### Physiological Features

The breathing rate was measured using a microphone attached to a headset that recorded the exhalations of the participants in a Waveform Audio File Format. To assess whether participants were able to breathe with a proper breathing technique during the exercise, the audio signal was visually inspected to count the number of exhalations. As it is generally advised to breathe in line with the movement, 10 exhalations were considered a perfect breathing rhythm. Continuous heart rate was monitored by means of a chest strap (RS800CX, Polar Electro) throughout the whole experiment. BP (Mobilograph) was monitored at the end of each set.

#### Self-Reported Features

##### Perceived Exertion

The Borg 0-10 category ratio scale (Borg CR-10) was used to assess the amount of perceived physical exertion in the participants [20]. The scale has been validated for use in resistance exercise [21]. The linear scale ranges from *nothing at all* to *hard* to *very very hard (maximal)*. For this study, a Dutch translation of the scale was used. After each set, participants were asked to rate their overall effort by choosing any number on the scale, allowing ratings in between numbers as well. In addition, the subjective comments of participants were gathered to evaluate the potential of technology-enabled feedback and possible implications for future use. After the exercise session was completed, a semistructured interview was conducted, including questions about participants’ experience regarding the type of feedback received during the 3 resistance training exercises.

##### Focus of Attention

The focus of attention of the participants was measured using a 10-point scale, ranging from 0 complete dissociation (external thoughts, daydreaming, environment, and singing songs) to 10 complete association (internal thoughts, how body feels, breathing, and muscles soreness). This one-item scale proved to be a valid and effective measure of attention strategies during effortful physical activity in previous research [22].

##### Motivation

Two subscales of the intrinsic motivation inventory (IMI) scale, perceived competence and interest/enjoyment, were used as measures of motivation. The other 4 subscales (effort, value/usefulness, felt pressure and tension, and perceived control/choice) were not used as they were deemed redundant with other measures of this study or not applicable. The interest/enjoyment and perceived competence subscale included 7 and 5 items, respectively. Responses were given on a 7-item Likert-type rating scale ranging from *not at all true* to *very true*. Negatively phrased questions were reversed for analysis. Item questions were translated to Dutch to increase the understandability of the questionnaire. A reliability analysis was carried out on the items of both subscales. Cronbach alpha showed the questionnaire to reach acceptable reliability (α=.871 for interest/enjoyment and α=.937 for perceived competence). Dropping any item would reduce alpha, so all items were retained. The IMI has also been previously validated in a sports setting by McAuley et al [23].

### Procedure

The study laboratory closely resembled a living room environment, which benefits the external validity of the study. Before continuing with the study, participants’ BP was checked to ensure that it was safe to proceed. As caffeine, nicotine, alcohol, or recreational drugs may influence BP, it was communicated to participants upfront to refrain from them for at least two hours before the test. Baseline BP levels were measured, and participants who did not exceed the upper limit for prehypertension (<139/89 mm Hg) could continue.

At the beginning of each exercise, a human kinetic technologist demonstrated proper execution. Subsequently, participants were asked to evaluate different weights using a Borg-scale in 3 repetitions. This was done until a weight was found, which participants rated as a 3 on the scale, which is considered a moderate intensity load. Then, the participants stood in front of the Kinect and put on a headset with the microphone. During the experiment, participants were asked to perform 3 different resistance exercises, where each exercise consisted of 3 sets of 10 repetitions, each exercise using a different feedback condition. Participants were instructed to indicate if the load was too high to adjust it and were also informed of their freedom to withdraw from the study at any time.

**Figure 3:**
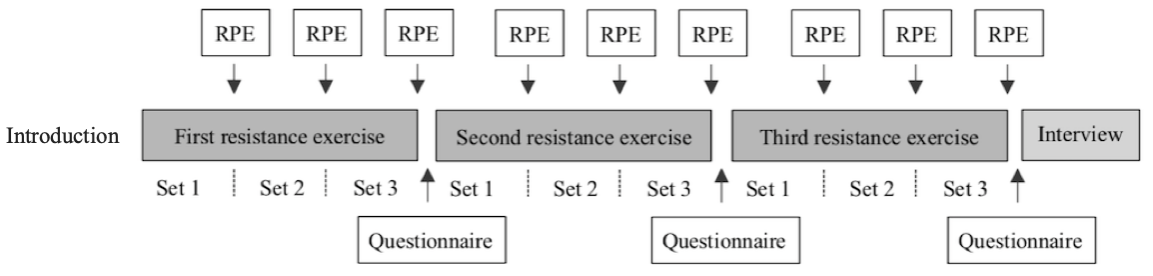
Study procedure. RPE: rating of perceived exertion.

After each set, participants were instructed to take place on a couch, where they were asked to indicate their rating of perceived exertion (RPE) for the set that was just completed. In addition, their BP was measured to ensure that it stayed within safe bounds. According to the ACSM, exercise should be stopped when BP exceeds 200 mm Hg systolic or 110 mm Hg diastolic, but as an additional precaution, the safety bound was set to <180/105 mm Hg. None of the participants reached this bound. After the exercise was completed (ie, after 3 sets), they were asked to fill out a questionnaire about their perceived competence, interest/enjoyment, and attention regarding the just finished exercise while a cup of water was served. Finally, after completion of the entire exercise session, the subjective experience of the participants was measured using a semistructured interview (Figure 3).

### Statistical Analysis

**Figure 4:**
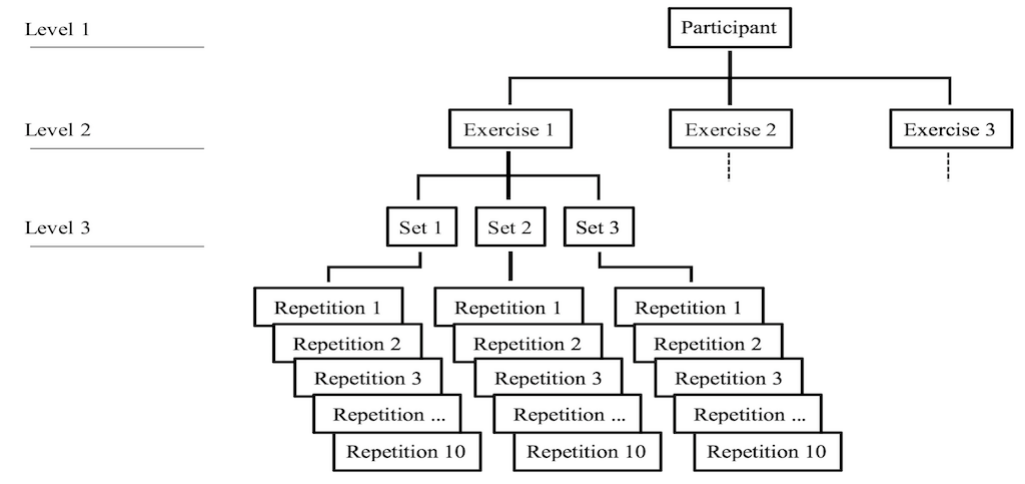
Hierarchical model.

To compare feedback and control conditions on measures of exercise performance and perceptions of exertion, a linear mixed effects regression (LMER) model was used for analysis, as observations on individuals were nested within higher-level groups (Figure 4). Compared with a more traditional approach with repeated-measures analysis of variance (ANOVA) analysis, LMER allows controlling for the variance associated with random factors without data aggregation.

Concentric/eccentric contraction times were compared on the repetition level, whereas concentric/eccentric end points, as well as respiration and RPE, were studied at the set level. To deal with nonindependence, the levels *participant* and *exercise type* were added as random factors. The software package LMER in R [24] was used to conduct the linear mixed effects analysis, where *P* values for the regression coefficients beta verbal, (β_V_), beta A/S (β_A/S_), beta repetition (β_rep_), and beta ambient light/sonification (β_set*A/S_) were obtained with the LMER test package [25].

Furthermore, to examine differences between feedback conditions on measures of attention, perceived competence, and intrinsic motivation, either a 1-way repeated-measures ANOVA test (in case the normality assumption was satisfied) or a nonparametric Friedman test (in case the normality assumption was violated) was used for analysis.

## Results

Descriptive statistics of the contraction times and contraction end points are presented in Table 2. Visual inspection of residual plots revealed a deviation from homoscedasticity and normality for both concentric and eccentric repetition times; therefore, a log10 transformation was applied to the dependent variable before analysis. To investigate whether there was a learning effect over the number of sets and repetitions for each of the feedback conditions, interaction effects with sets and repetitions were included.

**Table 2:** Descriptive statistics of all behavioral measures for each feedback type.

Contraction metrics	Control, mean (SD)	Verbal, mean (SD)	Ambient lighting and sonification, mean (SD)
Concentric contraction time (second)	2.17 (0.72)	2.76 (0.66)	2.48 (0.75)
Eccentric contraction time (second)	2.69 (0.91)	3.09 (0.75)	2.92 (1.05)
Concentric endpoint variation (mm)	17.93 (9.75)	19.66 (13.74)	15.64 (8.31)
Eccentric endpoint variation (mm)	10.70 (6.89)	12.94 (12.05)	12.77 (8.07)

### Concentric Contraction Time

**Figure 5:**
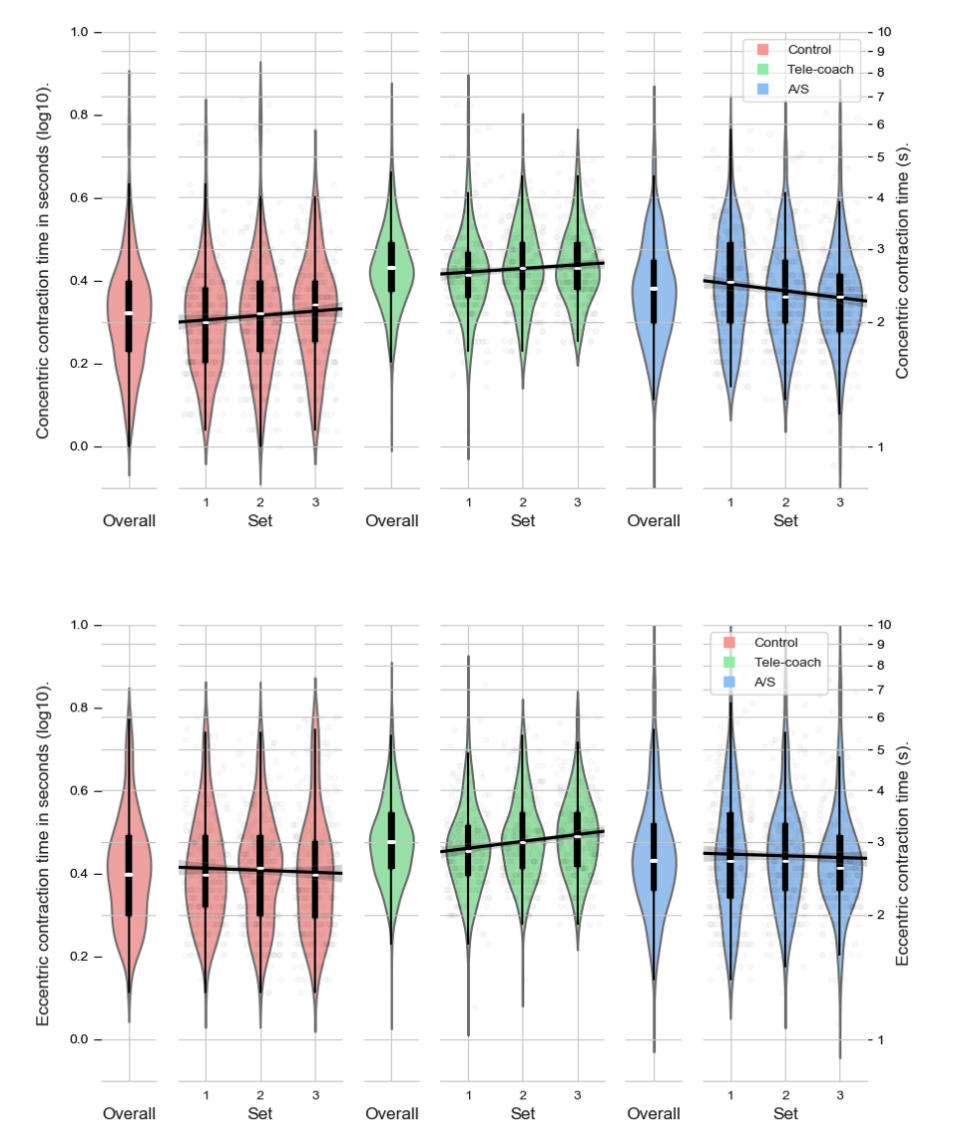
Distributions of concentric (top) and eccentric (bottom) contraction times, colored by condition, and reported per set. A/S: ambient light and sonification.

Violin plots of the concentric contraction time for each feedback condition are shown in Figure 5. To examine the effects of feedback on concentric contraction time, a series of linear mixed effects models was fitted using maximum likelihood estimation on log-transformed concentric contraction times. The model for concentric contraction time is shown in Table 3. Compared with the control condition (mean 2.17, SD 0.72), concentric contraction times were significantly higher and closer to the target of 3 seconds in the verbal feedback (mean 2.76, SD 0.66; β_V_=.124; *P*<.001) and A/S feedback condition (mean 2.48, SD 0.75; β_A/S_=.066; *P*<.001). Subsequent *sets* were performed a little slower (β_set_=.011, *P*<.001), and within a set, the pace of concentric contractions increased (β_rep_=−.005; *P*<.001; Figure 5). However, in the case of A/S feedback, concentric contraction times actually decreased from set 1 to set 3 (β_set*A/S_=−.029; *P*<.001; Figure 5).

**Table 3:** Multi-level mixed model parameters.

Modeled parameter^a^ and key characteristics of modeled parameter		Beta	SE (beta)	*t* test value
**Concentric contractions**				
	Intercept	.315	.023	14.02^b^
	Verbal	.124	.005	25.45^b^
	A/S^c^	.066	.005	13.95^b^
	Set	.011	.004	2.87^d^
	Rep	−.005	.001	−4.53^b^
	Set x^e^ verbal	−.001	.006	−0.25
	Set x A/S	−.029	.006	−5.12^b^
	Rep x verbal	−.001	.002	−0.77
	Rep x A/S	−.000	.002	−0.53
**Eccentric contractions**				
	Intercept	.408	.033	12.52^b^
	Verbal	.082	.005	17.42^b^
	A/S	.038	.005	8.33^b^
	Set	−.005	.004	−1.25
	Rep	−.002	.001	−1.93
	Set x Verbal	.020	.005	3.66^b^
	Set x A/S	−.001	.005	−0.19
**Concentric end points**				
	Intercept	17.41	2.53	6.87^d^
	Verbal	1.73	.40	4.31^b^
	A/S	−1.99	.39	−5.07^b^
	Set	−1.35	.33	−4.03^b^
	Set x Verbal	−3.19	.48	−6.72^b^
**Eccentric end points**				
	Intercept	10.78	3.98	2.71
	Verbal	3.89	.29	13.54^b^
	A/S	2.02	.28	7.19^b^
	Set	−0.75	.24	−3.17^d^
	Set x Verbal	1.28	.34	3.76^b^
	Set x AS	−0.13	.34	−0.38
**Respiration**				
	Intercept	11.10	.54	20.42^b^
	Verbal	1.86	.13	14.22^b^
	A/S	3.51	.13	27.03^b^
	Set	−.04	.11	−0.36
	Set x Verbal	−.46	.16	−2.93^d^
	Set x AS	−.65	.16	−4.20^b^
**Perceived exertion**				
	Intercept	3.65	.24	14.95^b^
	Verbal	.04	.03	1.37
	A/S	−.31	.03	−11.72^b^
	Set	.15	.02	6.43^b^
	Set x Verbal	.02	.03	0.72
	Set x AS	−.01	.03	−0.45

^a^For each of the conditions (verbal, A/S, and intercept representing the control condition), slope estimates (beta), their variation across participants (SE), and the *t* test value are given.

^b^*P*<.001.

^c^A/S: ambient lighting and sonification.

^d^*P*<.01.

^e^x: interactions between effects.

### Eccentric Contraction Time

As shown in Figure 5, eccentric contraction times were relative to the control condition (mean 2.69, SD 0.91), significantly higher and closer to the target of 3 seconds in the verbal feedback condition (mean 3.09, SD 0.75; β_V_=.082; *P*<.001) and A/S condition (mean 2.92, SD 1.05; β_A/S_=.038, *P*<.001). In the case of verbal feedback, later *sets* were performed a little slower (β_V_=.020; *P*<.001), but in the control (β_set_=−.005; *P*=.39) and A/S feedback (α_set*A/S_=−.001, *P*=.85), the pace of eccentric contractions between sets remained constant (Figure 5). Furthermore, an effect of repetitions was found (β_rep_=−.002; *P*<.001), eccentric contraction times were consistent within each set, as can be seen in Figure 5.

The V (ie, verbal) versus A/S model revealed that eccentric contraction times were significantly lower in the A/S feedback condition (mean 2.92, SD 1.05) compared with the verbal feedback condition (mean 3.09, SD 0.75; β_V vs A/S_=−.044; *P*<.001). However, from Figure 5, it can be tentatively concluded that both feedback types resulted in comparable support in reaching eccentric contraction times close to the instructed pace of 3 seconds.

### Concentric End Point Variations

**Figure 6:**
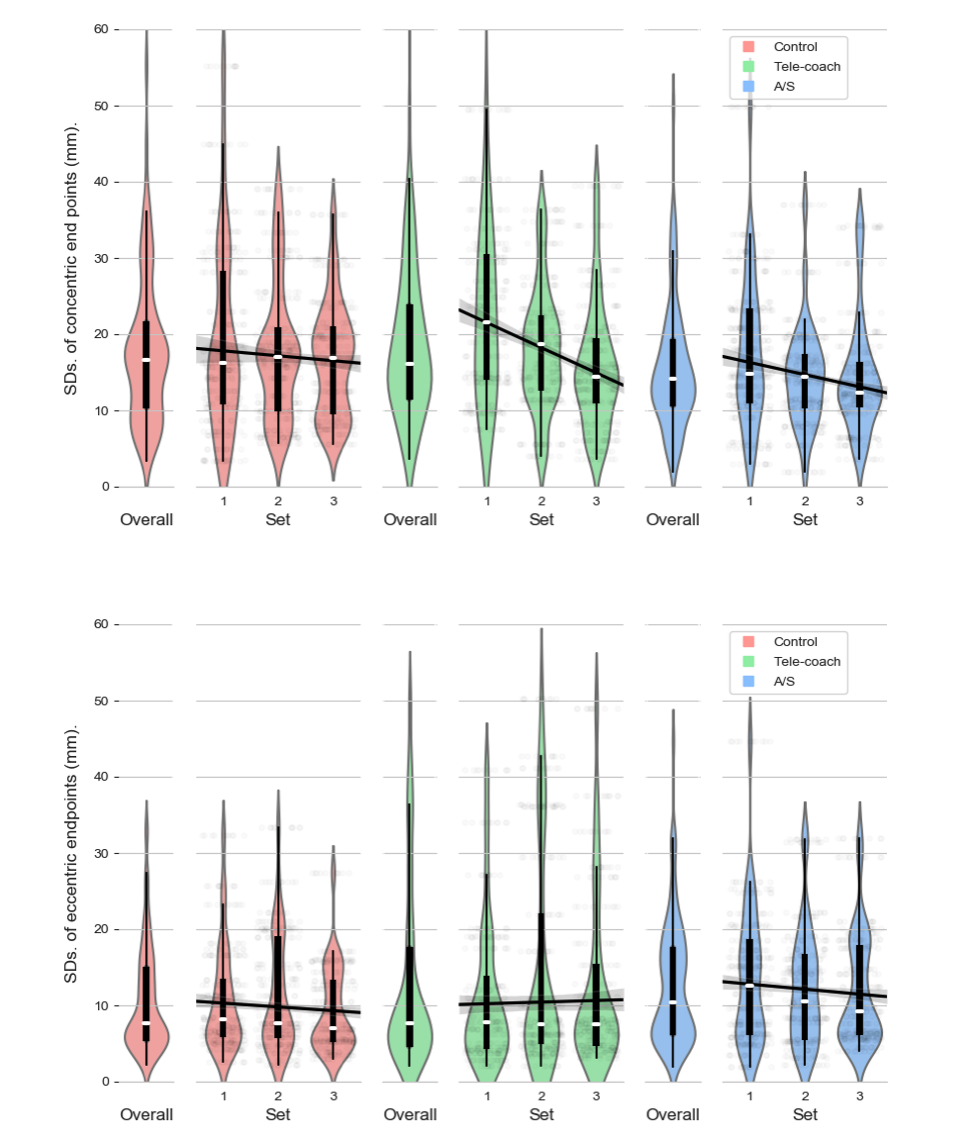
Distributions of the variations (standard deviation) in concentric (top) and eccentric (bottom) contraction endpoints colored by condition and reported per set. A/S: ambient light and sonification.

The model for concentric end point variations is shown in Table 3. As can be seen in Figure 6, compared with the control condition (mean 17.94, SD 9.75), the spread of concentric end points per set was significantly higher in the verbal feedback condition (mean 19.66, SD 13.74; β_V_=1.73, *P*<.001) but lower in the A/S feedback condition (mean 15.64, SD 8.31; β_A/S_=−1.99, *P*<.001). Although on average, participants performed best in the A/S feedback condition over time (Figure 6), there was a stronger decrease in concentric end point variation in the verbal feedback condition (β_set*V_=−3.19; *P*<.001) compared with the other 2 conditions (β_set_=−1.35, *P*<.001 and β_set*A/S_=−.22, *P*=.64).

### Eccentric End Point Variations

Table 3 shows the results of the linear mixed effects analysis for eccentric end points. Relative to the control condition (mean 10.60, SD 6.89), as shown in Figure 6, variations of eccentric end points per set were significantly higher in the verbal feedback condition (mean 12.94, SD 12.05; β_V_=3.89; *P*<.001) and A/S feedback condition (mean 12.77, SD 8.07; β_A/S_=2.02; *P*<.001). Eccentric end point variation decreased for every next set in the control and to a similar extent in the A/S feedback condition (β_set_=−.75, β_set*V_=−.13; *P*=.70). However, in the case of verbal feedback, the variation in eccentric end points actually increased from set 1 to set 3 (β_V_=1.28; *P*<.001), as can also be seen in Figure 6.

### Respiration

**Figure 7:**
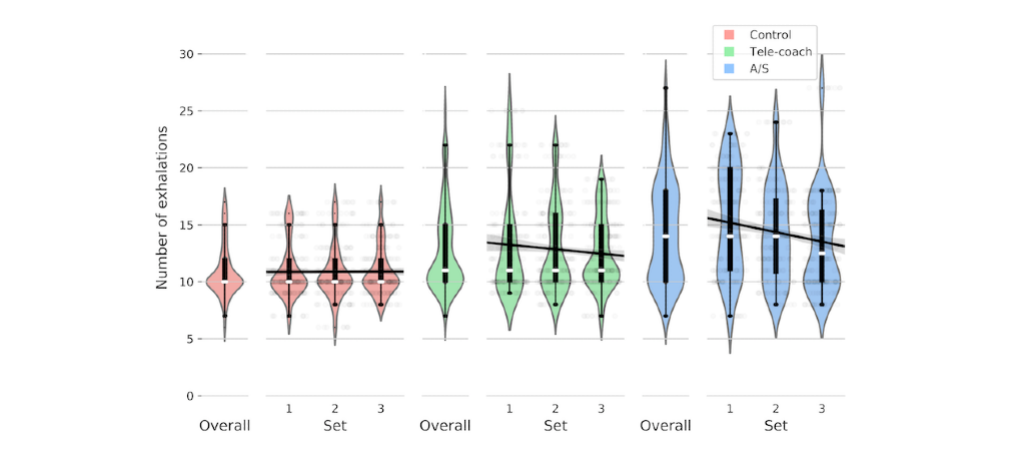
Distribution of total number of exhalations recorded over a set, colored by the condition. A/S: ambient light and sonification.

The results of the linear mixed effects analysis for respiration are displayed in Table 3. As Figure 7 shows, the number of participants’ exhalations significantly increased from the control condition (mean 10.88, SD 2.28 exhalations per set) to the verbal feedback condition (mean 12.83, SD 3.86 exhalations per set; β_V_=1.86; *P*<.001) as well as to the A/S feedback condition (mean 14.29, SD 4.46 exhalations per set; β_A/S_ =3.51; *P*<.001). Furthermore, from Figure 7, it can be tentatively concluded that the number of attempts of Valsalva maneuver did not differ among feedback conditions. Participants did improve over time (Figure 7), showing a decrease in respiration rate with later sets in both the verbal feedback condition (β_set*V_=−.46; *P*<.001) and A/S feedback condition (β_set*A/S_=−.65; *P*<.001). As there was no main effect of the *set*, the rate of respiration remained constant in the control condition (β_set_=−.04; *P*=.72).

### Perceived Competence

**Figure 8:**
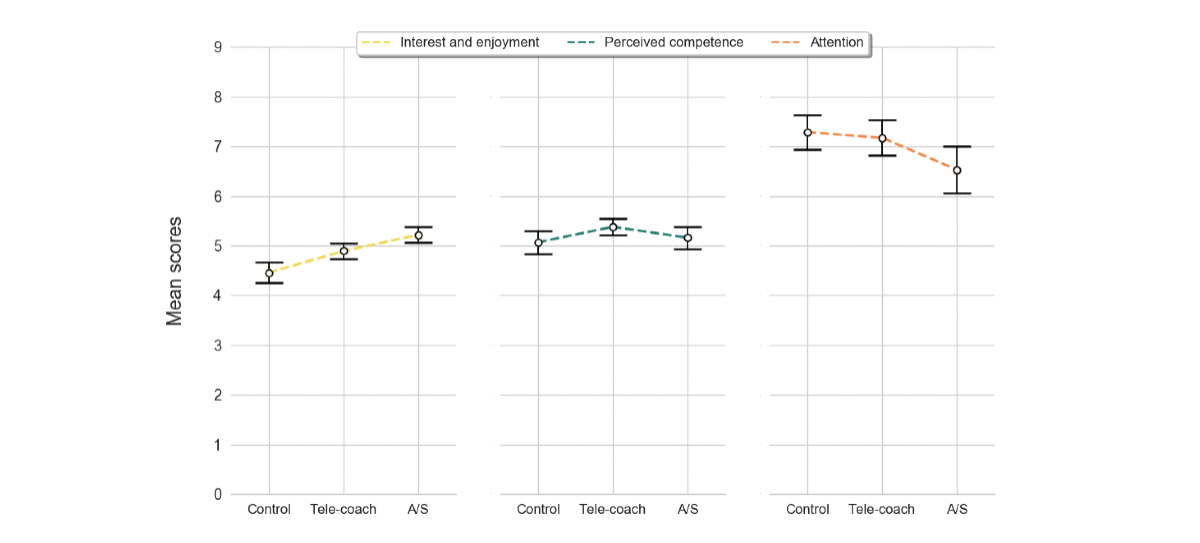
Distributions (mean and SE bars) of scores for psychophysiological measures from left to right: interest/enjoyment, perceived competence, and attention, reported per set. A/S: ambient light and sonification.

The perceived competence scores are shown in Figure 8. The sonification condition was not normally distributed; therefore, a nonparametric Friedman test was used to investigate intervention effects. There was no significant difference in perceived competence between feedback conditions (χ^2^_2_=0.6; *P*=.75).

### Interest/Enjoyment

Interest and enjoyment are shown per condition in Figure 8. A 1-way repeated-measures ANOVA was used to examine whether there was a significant difference in interest/enjoyment among feedback conditions. The Mauchly test of sphericity indicated that the assumption of sphericity was violated (χ^2^_2_=9.74; *P*=.01); hence, the Huyhn-Feldt Epsilon correction was used. A significant effect of feedback type on interest/enjoyment was found (*F*_1.666,58.310_=12.380; *P*<.001; partial η^2^=0.261). Post-hoc pairwise comparisons using the Bonferroni correction revealed that verbal feedback elicited an increase in interest/enjoyment compared with no feedback (mean 4.89, SD 0.95 vs mean 4.46, SD 1.25, respectively), which was significant (*P*=.03). Exercising with A/S feedback increased interest/enjoyment the most (mean 5.22, SD 0.93), which was significantly different from no feedback (*P*<.001) and verbal feedback (*P*=.05).

### Focus of Attention

The focus of attention is shown in Figure 8. On average, the attention of the participants was more diverted in the A/S feedback condition (mean 6.53, SD 2.81) than in the control (mean 7.29, SD 2.05) and verbal feedback condition (mean 7.17, SD 2.11). However, a Friedman test (the normality assumption was violated for all feedback types) indicated that attention scores were not statistically different among feedback conditions (χ^2^_2_=0.698; *P*=.71).

### Rating of Perceived Exertion

**Figure 9:**
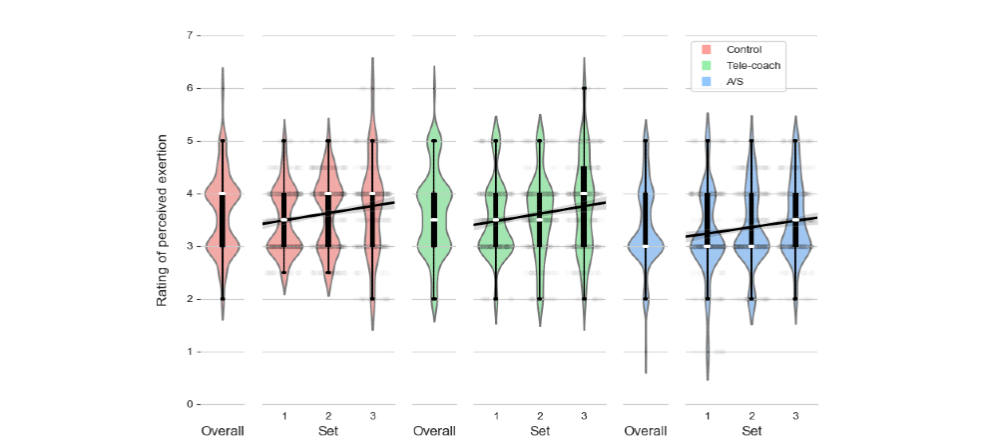
RPE per set, colored for each of the 3 conditions. A/S: ambient light and sonification; RPE: rating of perceived exertion.

Results of the linear mixed effects analysis for RPE are presented in Table 3. As can be seen in Figure 9, participants rated their perceived level of exertion to be significantly lower when the exercise was accompanied by A/S feedback (mean 3.37, SD 0.78; β_A/S_=−.31; *P*<.001) compared with the control (mean 3.64, SD 0.76) and verbal feedback (mean 3.64, SD 0.82; β_V vs A/S_=−.36; *P*<.001). As shown in Figure 9, perceptions of effort increased from set 1 to set 3 (β_set_=.15; *P*<.001), and this increase was the same in all conditions (β_set*V_=.02, *P*=.47 and β_set*A/S_=−.01, *P*=.65).

### User Experience

Of the 34 participants, 16 indicated that they preferred the verbal condition, 15 favored the ambient light sonification feedback, and 3 participants indicated they preferred to exercise without the addition of feedback. One participant did not make a clear statement regarding the condition preference.

## Discussion

### Previous Research

Previous research has used sonification to improve movement in physical activity [26,27]. However, its research base remains scarce, and its application in resistance training is limited. Therefore, this study aimed to investigate its potential as a feedback intervention in the home environment to improve resistance training performance. A feedback system was developed in which the left-hand movement of participants was analyzed and sonified for the purpose of improving exercise performance and compared with a control condition where no feedback is provided and a verbal condition that represents human verbal feedback.

### Effect of Ambient Lighting and Sonification Feedback on Exercise Performance

It was found that, in line with our hypothesis, A/S feedback resulted in more consistent concentric and eccentric contraction times with what had been instructed compared with the control. However, compared with the verbal condition, A/S feedback offered less support for concentric contraction times, but to a similar extent for eccentric contraction times. Thus, when people exercised without feedback, they were inclined to go faster than what was instructed, but with the support of verbal feedback as well as A/S feedback, their exercise pace could be corrected. When inspecting the results at the set level, it can be noticed that A/S feedback becomes significantly less effective in providing support for concentric contraction times over the 3 sets. Interview results revealed that about one-third of participants stated that they disliked the sonification used, such as the change in pitch, melody, and corrective earcon, which might explain the decline in performance over sets in this condition. Subsequent research may look into how the sound aesthetics of sonification can be improved and/or personalized to individual preferences.

It was also hypothesized that A/S feedback would support participants with a proper range of motion. The results are mixed, showing more consistent concentric contraction times in this condition, but less consistency for eccentric end points compared with verbal and control conditions. However, the magnitude of the differences between feedback conditions was small, suggesting that people generally have no difficulty in finding the right range of motion.

Moraveji et al [28] demonstrated that people can also adapt their breathing to a visual stimulus without requiring their full attention. However, whether pulsating light can also be effective without requiring people’s full attention was not known. The results did not provide evidence for this because the respiration rates of the participants were higher in both feedback conditions than in the control condition, in which their breathing technique adhered most to exercise recommendations. Increased arousal during exercise may reduce the attention allocated to background lighting. The majority of participants mentioned that they found the ambient light unsupportive or did not notice them, where some even mentioned that their breathing technique seemed to worsen because of the light. The results of this study further showed that the respiration rates of the participants were especially high for the first set but started to decrease with subsequent sets. This might indicate a habituation effect to the A/S feedback system that was not accounted for in their first interaction with the system.

### Effect of Ambient Lighting and Sonification Feedback on Perceived Competence and Intrinsic Motivation

Past research suggests that fostering people’s perception of competence can result in higher quality motivations, which in turn have been found to positively predict exercise adaptation and maintenance [29,30]. It is further suggested that this can be achieved by providing positive and corrective (verbal) feedback [31,32]. However, whether this can also be accomplished through positive and corrective nonspeech feedback (ie, A/S feedback), in a resistance training situation, has not yet been investigated. In contrast with our hypothesis, the results showed that neither verbal nor A/S feedback conditions had a significant effect on perceived competence. Thus, after 3 sets of resistance training with verbal or A/S feedback conditions, participants did not feel more competent than the control in performing the exercise correctly. It was found that participants on average had high scores on perceived competence, regardless of the feedback they received. A possible reason for this is that the 3 exercises selected for this research were easy to carry out or that the weight used to exercise with was not challenging enough. According to Deci et al [33], such feedback promotes competence when the activity provides an optimal challenge. The interview results supported this as the exercises were generally considered to be easy. A more speculative explanation would be that feedback was perceived as negative, which may hamper the positive effects on competence. It might have been that to build confidence, participants needed more time with the A/S feedback system.

The results indicate that people reported to be significantly more intrinsically motivated for the verbal and A/S feedback condition compared with the control, and the A/S feedback condition had a larger effect size than the verbal condition.

### Effect of Ambient Lighting and Sonification Feedback on Attentional Focus and Rating of Perceived Exertion

Previous research suggested that auditory and visual stimuli, often in the form of music or video, can be effective dissociative strategies to distract people’s attention from internal sensations that may also reduce perceptions of effort [22,34]. There is a clear trend that people in the A/S condition have a more dissociative focus than in the verbal and control conditions. Furthermore, the results indicated that when participants were presented with feedback in both sensory modalities, they reported a significantly lower RPE when compared with the other conditions, even though the initial load was comparable. Thus, it appears that when feedback is presented in both the auditory and visual sensory modality, participants may be more distracted from internal stimuli and, at the same time, report a lower RPE. These results are in line with the effects of music and video on effort [34]. Further research is warranted to examine whether lowering perceived exertion during resistance training in response to dissociative attentional stimuli (ie, feedback) has implications for resistance training adherence.

### Comparison to Related Work

In the sonification workshop of Schaffert and Effenberg [13], it was observed that rowing athletes cared primarily about the functional aspect of the sound, and not necessarily its esthetics. This is not in line with the findings in this work, where a considerable subgroup did not enjoy the sonification. This could possibly be because athletes care more about the performance quality of exercises and thus are willing to listen to unrefined sounds if these can aid them in performing better. Both findings were obtained through interviews. A quantitative comparison between sound esthetics might be more conclusive. Yang and Hunt [15] used sonification to guide the movements of bicep curls and achieved similar results as in this study, showing that the feedback has a positive effect on the pacing of the movement. They also showed a higher enjoyment when the feedback system was used in comparison with the control, similar to the increased enjoyment measured with the IMI in this study.

As for ambient light as a mechanism to guide the pace of breathing, previous studies have shown that people are able to synchronize their breathing to visual cues, such as during radiotherapy [16], to reduce people’s motion. This is not what was observed in this study. However, most of these studies were set up so that the full attention of the participants was focused on the visual cue. Findings by Brandt [35] showed that participants do not synchronize their pace of breathing well to the ambient light when not explicitly instructed to do so. The increase in arousal during exercise in this study might have also limited the attention pool available to focus on the ambient light and thus resulted in a similar effect, where people did not respond to the ambient light.

### Limitations and Future Research

There are limitations to this study. It could be that participants may have mixed up the ambient light and sonification feedback, trying to align breathing to sonic feedback instead of visual stimuli, and vice versa. Interview results suggest that this was unlikely, as most participants clearly noted that the visual stimuli were not supportive for breathing, suggesting that participants knew how to interpret the stimuli, but future research is warranted to investigate the stimuli separately. Next, the limited challenge associated with the light weights might have influenced the measure of perceived competence, and future research should study how the measures are affected under different intensity levels. Finally, the study was limited in duration and may have been sufficient to account for habituation effects to the system, and longitudinal aspects of a resistance exercise intervention are unknown, both of which provide opportunities for future study.

### Conclusions

An ambient lighting/sonification (A/S) feedback system was evaluated for its ability to support individuals in performing resistance exercise according to guidelines in a home setting. It was contrasted against a control condition in which participants did not receive feedback and a reference condition in which a human verbally provided feedback. Although the verbal condition was best at enhancing concentric contraction times, the developed concept of A/S feedback also succeeded in improving participants’ contraction times compared with the control. Furthermore, it improved the range of motion, where it improved concentric contraction end points more than verbal and control, and eccentric contraction end points more than control only. Ambient light turned out to be unsupportive of a proper breathing technique during resistance exercise. With respect to psychological determinants of physical activity, both A/S feedback and verbal conditions failed to promote perceptions of competence. Participants did, however, report higher levels of intrinsic motivation for A/S compared with both verbal and control conditions. Finally, it was found that the A/S feedback resulted in a trend where participants reported having a more dissociative focus while also reporting a significantly lower perception of effort. It would be interesting to test in future studies whether a combination of auditory and visual feedback may be used during resistance training to lower perceptions of effort, which could potentially increase exercise adherence. In this study, A/S feedback assistance seemed to improve exercise execution and psychosocial attitude of individuals who were normotensive and prehypertensive when performing a single session of home-based resistance exercise in comparison to using no feedback at all, but there was no clear advantage over a human tele-coach providing verbal feedback.

## Appendix

Multimedia Appendix 1 Permutations diagram.

## Abbreviations

A/S: ambient light and sonification
ACSM: American College of Sports Medicine
ANOVA: analysis of variance
BP: blood pressure
IMI: intrinsic motivation inventory
LMER: linear mixed effects regression
RPE: rating of perceived exertion
