# Supplementary figures and images for: Assisting Home-Based Resistance Training for Normotensive and Prehypertensive Individuals Using Ambient Lighting and Sonification Feedback: Sensor-Based System Evaluation
Source: JMIR Cardio. 2020 Jun 29;4(1):e16354. doi: 10.2196/16354 (PMC7367528; doi:10.2196/16354)

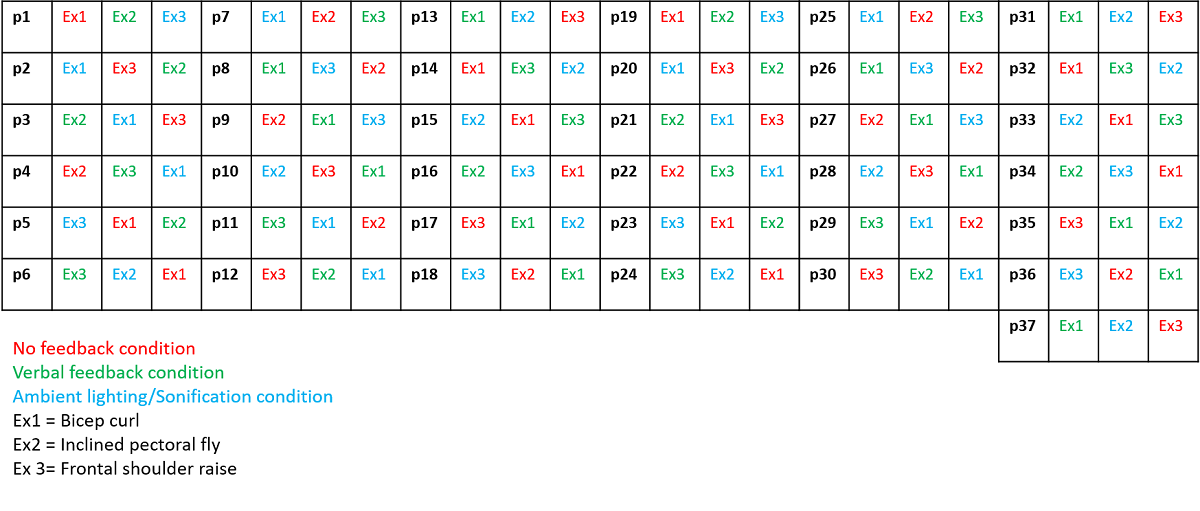

Supplement: Multimedia Appendix 1 [file cardio_v4i1e16354_app1.png]
